# Does the chronically ill population in the Netherlands switch their health insurer as often as the general population? Empirical evidence from a nationwide survey study

**DOI:** 10.1186/s12913-020-05228-z

**Published:** 2020-05-05

**Authors:** Wouter van der Schors, Anne E. M. Brabers, Judith D. De Jong

**Affiliations:** 1 Nivel, the Netherlands Institute for Health Services Research, PO Box 1568, 3500 BN Utrecht, the Netherlands; 2 Erasmus University Rotterdam, PO Box 1738, 3000 DR Rotterdam, the Netherlands; 3 Maastricht University, PO Box 616, 6200 MD Maastricht, the Netherlands

**Keywords:** Switching, Health insurance, Managed competition, Chronic disease, Choice, Consumer mobility, Health system reform

## Abstract

**Background:**

Consumer mobility is an important aspect of a health insurance system based on managed competition. Both the general population and insured with a chronic illness should enjoy an equal opportunity to switch their insurer every year. We studied possible differences in the rates of switching between these two groups in the Netherlands.

**Methods:**

A structured questionnaire was sent to 1500 members of Nivel’s Dutch Health Care Consumer Panel (response rate: 47%) and to 1911 chronically ill members of the National Panel of the Chronically ill and Disabled (response rate: 84%) in February 2016. Associations between switching and background characteristics were estimated using logistic regression analyses with interaction effects.

**Results:**

In general, we did not find significant differences in switching rates between the general population and chronically ill population. However, a combination of the population and background characteristics demonstrated that young insured with a chronic illness switched significantly less often than young insured from the general population (1% versus 17%).

**Conclusions:**

Our results demonstrated that the group of young people with a chronic illness is less inclined to switch insurer. This observation suggests that this group might either face difficulties or barriers which prevents them from switching, or that they experience a high level of satisfaction with their current insurer. Further research should therefore focus on unravelling the mechanisms which explain the differences in switching rates.

## Background

Over the last decade many OECD countries have implemented elements of managed competition in their health care systems [1, 2]. For the Netherlands, a prominent example for managed competition, the health care reform in 2006 is recognised as an important structural health care reform [3]. This reform played a major role in the shift from supply-side government regulation to managed competition. It was based on a concept developed by Enthoven in 1978 [3–5].

The Dutch government’s reforms of the health insurance market was aimed at solving several structural problems, for instance the growth in health expenditures, the lack of competition, and the lack of pressure on suppliers to achieve better performance [3, 6]. Based on Enthoven his concept of managed competition, the Dutch government formulated several goals for the Health Insurance Act (HIA). First, the HIA should lead to increased expediency and more freedom of choice for all insured. Second, it should decrease the government’s central control of health insurance provision. The third aim was that the HIA must guarantee good accessibility of care for all [7]. An important aspect of the HIA is the possibility to switch health insurer [8]. All insured are allowed to switch insurer or insurance plan every year, during an annual enrolment period. People can switch insurer for (1) the universal basic insurance, which has a government-determined content across all plans, (2) the supplementary insurance, or (3) for both. Those insured who are dissatisfied with their current plan or look for a plan that meets their preferences and needs better could opt for another insurer. People can for instance switch because of the premium, content of the supplementary insurance, premium discount for the voluntary deductible (See Table 1) and/or level of service. The assumption is that every insured person, whatever their characteristics, such as age or chronic illness, has the same opportunity to switch [9]. The insured would then, in theory, incentivize health insurers to meet their preferences regarding price, quality and contracted health care [10].

**Table 1:** Health insurance in The Netherlands [3]

Basic insurance:	
− *National government yearly determines the content of the basic insurance policies* − *The content is identical for every basic insurance* − *The basic insurance is compulsory for everyone living or working in The Netherlands aged 18 years and above* − *Health insurers are obliged to accept everyone regardless their age, health status and place of residence* − *Premium differentiation is prohibited* − *Insured have the possibility to switch once a year between health insurers* − *Distinction between policies with full reimbursement of all health care providers (reimbursement policy) or policies with partly reimbursement of the non-contracted health care providers (in kind policy)* − *Mandatory deductible of 385 euro in 2016. This does not apply to care that is reimbursed by any supplementary insurance, or for a few types of care in the basic insurance, such as GP care.* − *Possibility to choose for a voluntary additional deductible of 100, 200, 300, 400, 500 euros to decrease the monthly premium.* − *The basic insurance is an extensive package covering, among others, GP care, hospital care and maternity care*	
Supplementary insurance:	
− *The supplementary insurance covers, among others, physiotherapy for non-chronic illnesses, dental care and additional reimbursements for medicines* − *Voluntary, not necessarily taken out at the same insurer as the basic insurance* − *Insurers are not obliged to accept everyone* − *Premium differentiation is allowed for the supplementary insurance*	

Several safeguards were introduced in order to achieve an equal opportunity to switch health insurer, both for the general population and those insured suffering from a chronic illness (from here on mentioned as chronically ill population). These safeguards are outlined in Table 1. The underlying idea is that differences in nominal premium rates between health insurers reflect differences in efficiency and not differences in the insured population. Among others, these safeguards impose that health insurers are obliged to accept all applicants for a basic package, while this is not the case for the supplementary insurance. Moreover, for the basic package there is an obligation to apply community rating when calculating the nominal premium, thus accepting everyone for the same premium which implies that applying different premiums for different risks (risk rating) is forbidden for the same insurance policy. Risk equalization is used to compensate health insurers for differences in the risks present in their insured population. For instance, a health insurer with an overrepresentation of patients with high expenditures receives more compensation from a central fund. A well-functioning system of risk equalization should avoid health insurers to focus on attracting low-risk insured or try to cream-skim on quality in order to attract low-risk patients. The Dutch risk equalization formula is widely regarded as a sophisticated instrument. To a large extent, it mitigates the higher or lower expenditures caused by case-mix differences between health insurers [11]. However, recent research highlights shortcomings in risk equalization [12]. For instance, someone with a chronic condition as diabetes resulted in an average loss of 221 euros for the health insurer. Imperfect risk equalization may incentivize risk selection among health insurers [13].

In 2016, the year of this study, 25 different insurers were on the market offering together 61 different insurance policies. For all insurers the basic package has the same content but might differ in price (See Table 1). Although the purchaser side of the health insurance market is highly regulated by the government, health insurers are allowed to selectively contract health care providers which offer the best value for money ratio [14]. The government created more freedom to negotiate with health care providers, for instance by the major increase in freely negotiable hospital prices. This results in in-kind plans, for which not all providers are fully reimbursed. In practice, most of the healthcare providers are still contracted by most health insurers. If the combination of safeguards described in Table 1 work as intended, both groups should be equally attractive to health insurers and health insurers will feel little incentive to engage in risk selection [15]. Furthermore, there would be no barriers for those insured with a chronic illness to switch health insurer.

In this study, we will compare the rates of switching between the chronically ill population and the general population in 2016. Here, switching is defined as the switch from one insurer to another insurer. Making an adjustment to a plan with the same health insurer organization is not defined as switching in the current study. Switching between health insurers has been studied before, mainly in the Dutch setting [8–10, 16]. However, these studies show ambiguous results concerning the frequency of switching in the general population and the chronically ill population. One study showed a significantly lower switching rate between insurers among the chronically ill population in comparison to the healthy insured, however, this effect disappeared when corrected for age [9]. Three other studies found no significant differences between the two groups, but showed that the chronically ill population may experience barriers to switching [12, 13, 17]. Furthermore, research among the general population showed that younger people, more highly educated people, and people with a better perceived health condition, switch health insurer more often than older, lower educated and insured with a worse perceived health condition [18].

Our study aims to contribute to the current body of evidence on the switching behavior in three ways. First, to date the role of age, level of education and perceived health condition on the behaviour of switching is generally well established for the general population, but to a lesser extent for the chronically ill population. This research aims to address this paucity by establishing the relationship between the attributes and switching behaviour for both populations. Second, in the recent years, important steps have been taken by government bodies and health insurers to create equal possibilities to switch health insurer. Therefore, as earlier research is based on data from ten years ago, conducting a study based on up-to-date data is relevant in order to assess whether there are differences in switching rates. Third, the assessment of switching behaviour in the Dutch health insurance market provides a relevant case both for all countries with a health insurance market based on managed competition, and for all countries considering introducing such a system. Both should determine whether the chronically ill population switches to the same extent as the general population as this gives insight into whether the system works as intended.

The following two research questions will be answered in this article:

Q1: Are there differences in switching rates between chronically ill population and the general population?

Q2: What is the role of the background characteristics age, level of education and perceived health condition in explaining the switching rates for the chronically ill and general population?

### The switching rates for the chronically ill population and the general population

The main idea behind switching insurer is either that the insured will search for a new insurer because they are dissatisfied with the premium or quality of health care offered by their insurer, or because another health insurer offers a better option [10]. The insured are expected to search for information about price or quality in order to find a new health insurer. Information related to price is generally easier to find and compare than that related to quality [7, 19]. For the general population, it is easier to select a new health insurer since they will tend to use less health care and thus can focus more on differences in the insurance premium. By contrast, the chronically ill population will tend to use more care, and, in particular, more specialised care than the general population [20]. Therefore, it is important for the chronically ill to have a health insurer who provides a high quality of care and access to their preferred health care providers. Consequently, the chronically ill population is expected to consider both price, quality and contracted providers when choosing a new health insurer. They will thus have to invest more time, should consult more sources, and might face more uncertainty after a switch [21]. As such, it could be reasoned that the switching costs, in terms of time and effort, are expected to be higher for the chronically ill population than for the general population. On the other hand, it could be reasoned that a switch could also result in higher benefits for the chronically ill population, since their focus is related to aspects other than price. Furthermore, in comparison to the general population, the chronically ill population may feel more concerned that their claim for their supplementary insurance could be rejected when they switch. This might decrease the switching rates among the chronically ill population [22, 23]. Although the empirical literature does not demonstrate a consistent relationship, we hypothesize, based on the theory, that the general population will switch more often than the chronically ill population.

*Hypothesis 1: The general population switches more often than the chronically ill population.*

### The role of age, level of education, and perceived health condition

Empirical research demonstrates the positive relationship between the *level of education* and the propensity to switch between health insurers [24]. More highly educated people seem to switch more often than those with a lower education. The research on insurer choice demonstrates that a higher level of education leads to increased search activity compared to a lower level of education [25–27]. Second, more highly educated people have a better ability to reflect critically upon the information they have found, which makes it easier for them to make a choice [28].

Previous studies have also found that older insured people are less likely to switch in comparison with younger ones [9, 10, 29]. Being older is often accompanied with more uncertainty about the use of health care in the upcoming year. This can make the choice of a new health plan, in general, more complicated. For the older insured, choosing a new insurer may also be more complicated since they may be less capable of searching and processing information as fast compared to younger insured people, resulting in higher switching costs for the older insured [30, 31]. Higher switching costs decrease the chance of switching insurer. Furthermore, the older insured might switch less because they have often been insured for a long time with their current insurer. They are, therefore, familiar with the procedures of their current insurer and this will thus decrease the likelihood of switching since they want to avoid the regret and uncertainty which may accompany switching [32].

The role of *perceived health condition* on switching behaviour has similarities with the role of having a chronic disease but differs on the point that it refers to a more time dependent and self- reported measure instead of an objective measure as diagnosed by a general practitioner. For instance, people with a chronic illness can experience a good subjective health status. All combinations between both measures can occur. Here, the insured with a worse self-perceived health condition might look further than only a lower price offered by another health insurer and search for an insurer who matches their specific preferences. This search for information is more complicated and demands more time. We expect, therefore, that people with a better perceived health condition will switch more often than people with a worse perceived health condition.

*Hypothesis 2a: More highly educated people switch more often than lower educated people.*

*Hypothesis 2b: Younger people switch more often than older people.*

*Hypothesis 2c: People with a better self-perceived health condition switch more often than people with a lower self-perceived health condition.*

Having a chronic disease plays a major role in choosing a health insurer [33]. The demand for specific care makes the chronically ill population more focused upon quality over price. Therefore, it can be argued that possible variation in the switching rates could be ascribed mostly to the presence of a chronic disease instead of background characteristics. Thus, we expect the influence on switching rates of the background characteristics, age, level of education, and health condition, to be larger in the healthy population compared to the chronically ill.

*Hypothesis 3: Age, level of education, and perceived health condition, play a larger role among the general population compared to the chronically ill population*.

## Methods

### Data

Questionnaires were sent to members of two panels of Nivel (the Netherlands Institute for Health Services Research). One, the Dutch Health Care Consumer Panel (DHCCP) represents the general population while the other, the National Panel of the Chronically ill and Disabled (NPCD), represents the chronically ill population. In February 2016, a questionnaire was sent to 1500 members of the DHCCP. A total of 703 members returned the questionnaire (response rate: 47%). In April 2016, the same questions about switching were sent to 1911 members of the NPCD. A total of 1596 members returned the questionnaire (response rate: 84%). According to their previously stated preferences, members received a questionnaire by post or through the internet.

#### Dutch Health Care Consumer Panel

The DHCCP is a so-called access panel managed by Nivel. The panel aims to measure opinions on, and knowledge about, health care and the expectations and experiences with health care in the Netherlands [34]. In 2016, the panel consisted of approximately 12,000 members aged 18 years and older. Many background characteristics of the panel members are known, such as their age, gender and the highest level of education completed. New panel members are recruited on a regular basis to renew the panel. Renewal is necessary to make sure that members do not develop specific knowledge of specific health topics. It is not possible for people to enroll themselves in the DHCCP. Switching rates measured in the DHCCP are comparable with those among the whole Dutch population based on administrative data [35]. The DHCCP contains members with a chronic illness (see Table 2). In contrast to the diseases among the panel members of the NPCD which are diagnosed objectively, the chronic diseases among the DHCCP panel members are self-reported.

**Table 2:** Descriptive statistics

	General population (***N*** = 703)	Chronically ill population (***N*** = 1593)
**Age**		
18–39	21%	15%
40–64	50%	38%
65 years and older	29%	47%
**Education**		
Lower	15%	32%
Intermediate	54%	45%
Higher	31%	23%
**Perceived health condition**		
Bad	14%	34%
Good	53%	53%
Very good	33%	13%
**Sex**		
Male	50%	43%
Female	50%	57%
**Chronic illness**		
Yes	28%	100%
No	72%	0%

#### National Panel of the Chronically ill and Disabled

The NPCD was introduced by Nivel to give insight into patient perspectives on living with a chronic disease or disability in the Netherlands. In 2016, the panel consisted of approximately 3500 people above 15 years of age with one or more chronic diseases and/or with a physical disability. Participants with chronic diseases are selected from random samples of general practices in the Netherlands. The inclusion criteria are: a diagnosis of a somatic chronic disease, aged 15 years or older; not being permanently institutionalised; being aware of the diagnosis; not being terminally ill (life expectancy more than 6 months according to the general practitioner (GP)); being able mentally to participate; and having sufficient mastery of the Dutch language. Self-enrolment for the panel is not possible.

New panel members are recruited annually to replace participants who withdrew or had participated for the maximum term of four years. In addition to the self-reported demographic characteristics provided by the panel members, GPs provide medical information about the panel members, with the patients’ consent, about the chronic diseases diagnosed and the dates of the diagnoses. For this study, we selected panel members aged 18 years and over who had been diagnosed with at least one chronic disease.

### Privacy

The data collection is registered with the Dutch Data Protection Authority for both the DHCCP and the NPCD (nr. 1,262,949 and nr. 1,283,171, respectively). All data were collected and handled in accordance with the privacy protection guidelines of the Authority. Data were processed anonymously. A privacy regulation applies for both panels. According to Dutch legislation, neither obtaining informed consent, nor approval by a medical ethics committee, is mandatory for carrying out research in both panels [36].

### Measurements

#### Dependent variable: switching

Switching was measured by asking the respondents whether they had switched to another health insurer during the period of enrolment for 2016 (November 2015–February 2016). The question about switching (see Additional file 1 for exact question) is part of Nivel’s annual monitor about the switching behaviour of insured in the Netherlands. The original question consisted of five mutually exclusive categories, of which the respondents could choose only one. The five categories were 1) Not switched, not considered switching; 2) Not switched, considered switching; 3) Switched solely for the basic insurance; 4) Switched solely for the supplementary insurance, and; 5) Switched for both the basic and supplementary insurance. In our analysis switching is divided into two categories: the original options 1 and 2 were re-coded in not switched (coded as 0), while the original options 3, 4 and 5 were re-coded in switched (coded as 1). We created this combined variable for switching since the number of insured in both panels (NPCD and DHCCP) who switched for solely the basic insurance (*n* = 16), or solely the supplementary insurance (*n* = 4) was rather low.

#### Independent variables

We used the following independent variables to test our hypotheses: Age, 18–39 years (coded as 0), 40–64 years (coded as 1), and 65 years and older (coded as 2); level of education, low (coded as 0), intermediate (coded as 1), high (coded as 2); perceived health condition, very good/excellent (coded as 0), good (coded as 1), bad/poor (coded as 2); and, general population (DHCCP) (coded as 0), or chronically ill population (NPCD) (coded as 1). In our analyses, the chronically ill population thus refers to the membership of the NPCD. In addition, sex (0 = male, and 1 = female) was included as an independent variable.

### Analyses

#### Corrections

Before analysing the switching rates in the two panels, corrections were made to make the samples comparable. A weighting factor was calculated for the NPCD to make the sample representative of the Dutch population of people with a chronic illness, based on the proportion between the number of chronically ill people and the number of disabled people in the population [37]. In order to draw comparisons between both panels, we reweighted the chronically ill population to line up with the distribution of age, sex and education in the general population. This reduced the possibility of finding differences in the switching rates caused by differences in the distribution of background characteristics such as age, level of education and sex between the two panels. Therefore, a new variable for the chronically ill population was used for the combination of the three variables: age (three groups, 18–39, 40–64, 65 years and older); sex (male, female); and, level of education (lower, intermediate, higher) thus resulting in 18 separate weighting factors.

Table 2 demonstrates descriptive statistics showing chronically ill population among the NPCD panel are, in general, older and lower educated, compared with the general population. Furthermore, the uptake of supplementary insurance in both populations is similar (87% in the DHHCP and 86% in NPCD). In the general population, 28% reports a chronic illness. Among the group of DHCCP members with a chronic illness, 80% suffers from one self-reported illness, 18% from two and 2% from three or more illnesses.

#### Statistical analyses

First, descriptive analyses were performed to determine the switching rates for the different background characteristics among the two groups. Both weighted and unweighted switching rates were presented. Second, we performed a logistic regression analysis to test our hypotheses about the differences in the switching rates between the two populations and the role of background characteristics in this (Hypothesis 1, Hypotheses 2a-2c). A model was estimated in which switching functioned as the dependent variable, while age, sex, education, self-perceived health condition, and population, functioned as independent variables. Third, a second model was estimated to test whether the role of the independent variables, such as age, level of education, and perceived health condition, and the switching rates differ between the general population and the chronically ill (Hypothesis 3). To test this, interaction effects were added to our first model. To determine whether switching rates differed significantly between the two panels, odds ratios, marginal effects and *p*-values were reported. Significance levels were set at 5%. For all analyses, STATA version 14.0 was used.

## Results

Table 3 shows that 7% of the general population switched health insurer during the enrolment period in 2016. Among the chronically ill population, 4% switched insurer. When we examine the switching rates in the different age groups, we see that in the general population 17% of the insured aged between 18 and 39 years, switched. Among the chronically ill population, 1% between 18 and 39 years switched insurer. In both groups, more highly educated people and people with a very good or excellent perceived health condition reported the highest switching percentages.

**Table 3:** Weighted switching percentages (confidence intervals in brackets, unweighted percentages for the chronically ill population in italic) ^*a*^

		General population	Chronically ill population
**Switching rate**	Total	7% (5–9%)	4% (3–6%) *4%*
**Age**	18–39	17% (11–24%)	1% (0–4%) *2%*
	40–64	5% (3–7%)	6% (4–8%) *6%*
	65 years and older	4% (2–7%)	3% (2–6%) *3%*
**Education**	Lower	2% (0–8%)	5% (2–9%) *4%*
	Intermediate	6% (4–9%)	4% (3–5%) *4%*
	Higher	10% (7–15%)	5% (3–8%) *5%*
**Perceived health condition**	Bad	5% (2–12%)	5% (3–8%) *5%*
	Good	5% (3–8%)	4% (3–6%) *4%*
	Very good	10% (7–15%)	3% (1–7%) *3%*

^a^The weighted results are not presented for the general population. We matched the chronically ill population to the distribution of age, sex and education among the general population. As such, the weighted and unweighted results are equal for the general population

To test our Hypotheses 1 and 2a-2c, we performed a multivariate logistic regression analysis with switching as the dependent variable. The results in Table 4 demonstrate that the general population did not switch significantly more than the chronically ill population. Hypothesis 1 was therefore rejected. Furthermore, there was no difference in the switching rates between lower and higher educated people. Hypothesis 2a is therefore also rejected. The insured with a very good or excellent perceived health condition did not switch more often than the insured with a bad or poor perceived health condition. As such Hypothesis 2c is rejected. However, the results indicate that the younger insured switched more often than the older insured. As a sensitivity analysis, we performed a regression analysis for the switching rates in the DHHCP only (Additional file 2). There are no significant differences in switching rates between people with a self-reported chronic illness or without a chronic illness among the general population with regards to rates of switching, which is in accordance with the results found in the two-panel comparison.

**Table 4:** Multivariate logistic regression with switching as the dependent variable

		Model 1: Switching *Pseudo R2 = 0.03* *N = 1961*			Model 2: Switching with interaction terms *Pseudo R2 = 0.06* *N = 1961*		
**Switching (1 = yes. 0 = no)**		**Odds Ratio**	***P*-value**	**Marginal Effect**	**Odds Ratios**	***P*-value**	**Marginal Effect**
**Age**	18–39	Reference		0.08	Reference		0.06
	40–64	0.64	0.10	0.05	0,26	0.00*	0.05
	65 years and older	0.44	0.01*	0.04	0,25	0.00*	0.04
**Education**	Lower	Reference		0.05	Reference		0.04
	Intermediate	0.97	0.91	0.05	2,33	0.27	0.05
	Higher	1.45	0.24	0.06	3,38	0.12	0.06
**Perceived health condition**	Very good	Reference		0.06	Reference		0.05
	Good	0.78	0.36	0.04	0.66	0.22	0.04
	Bad	1.02	0.95	0.06	0,85	0.76	0.05
**Group**	General population	Reference		0.06	Reference		0.06
	Chronically ill population	0.67	0.08	0.04	0.16	0.10	0.04
**Sex**	Male	Reference		0.05	Reference		0.05
	Female	0.82	0.37	0.05	0.93	0.83	0.05
Age * group	18–39 * general population	–	–	–	Reference		0.13
	40–64 * chronically ill population	–	–	–	13.23	0.00*	0.06
	65 years and older * chronically ill population	–	–	–	7,07	0.01*	0.03
Education*group	Lower* general population	–	–	–	Reference	–	0,03
	Intermediate* chronically ill population	–	–	–	0,38	0,25	0,04
	Higher* chronically ill population	–	–	–	0,36	0,24	0,05
**Perceived health condition** * group	Very good*general population	–	–	–	Reference		0,07
	Good* chronically ill population	–	–	–	2,03	0,26	0,04
	Bad* chronically ill population	–	–	–	1,90	0,40	0,05
Sex*group	Male*general population	–	–	–	Reference		0,06
	Female*Chronically ill population	–	–	–	0,91	0,84	0,04
**Constant**		0.12	0.00*		0,09	0.00*	

*p < 0,05

Hypothesis 3 was tested by adding the interaction terms between all the independent variables (level of education, age, and perceived health condition) and population (general/chronically ill) to our first logistic regression model. As can be seen in Model 2 of Table 4, the interaction between age and group is significant. Further evaluation of the age effect in the chronically ill subgroup, achieved by changing the reference category (not shown in table), reveals that the switching rate among this subgroup is a little higher in the 40–64 group compared to the group of 18–39. In addition, further evaluation of the age effect demonstrates for the general population that switching decreases with age. Furthermore, it appears that the insured aged 18–39 years in the general population switch significantly more compared to the same age group among the chronically ill population.

## Discussion

This study investigated the differences in the behaviour of switching between the general population and the chronically ill population, the role the background characteristics, age, level of education and perceived health condition, played in this, and the possible different role of the background characteristics among these two groups.

### Switching insurer

The current study found that 7% of the general population, and 4% of the chronically ill, switched insurer in 2016. However, when we took age, sex, perceived health condition, and level of education into account, no significant differences in switching rates between the general population and chronically ill population were found. The general switching rates found in our study are particularly low compared to other consumer markets such as the switching rate found in the Dutch energy market (16%) in 2016 [38, 39]. However, they are in line with the switching rates found previously in the health insurance market in the Netherlands [10, 40, 41]. A possible explanation is that only a few insured are willing to consider switching health insurer each year [42].

We hypothesised that differences in the level of education, age and perceived health condition would result in differences in switching rates (Hypothesis 2a, 2b and 2c). The results showed only an effect with regards to age. The results are in line with the findings in previous studies that younger people switch more often than older people [9, 10]. In contrast to earlier findings, however, this study found no significant effect of the level of education (Hypothesis 2a). A possible explanation for this might be that a higher level of education might make it easier to process the information but does not tell whether more highly educated people search more information. The level of education as a measure for search behavior on its own, might be insufficient in explaining variation in switching.

The combination of the population and background characteristics (Hypothesis 3) demonstrates a difference in the age effect between the panels. The young insured with a chronic illness switched significantly less than young insured from the general population (1% versus 17%). They report the lowest rate of switching of all the groups compared in this study. One possible explanation for the low rate of switching amongst the youngest group of chronically ill people is the amount of time and effort which is taken up with choosing a health insurer. Having a chronic illness makes it necessary to select a health insurer which offers a good quality of care and access to the preferred doctor to safeguard continuity of care. This is especially the case when people are recently diagnosed with their chronic condition, which is more likely in the youngest age group. For this group, the choice might be thus more complicated and time consuming [7, 19]. Research into how people use their time in the Netherlands demonstrates that people aged between 18 and 40 years spend the most time on work and obligatory activities and have the least spare time of all age groups. This is especially true when people have children [43]. The combination of a complicated choice and time constraints might make the young chronically ill less inclined to switch. At the age of 18 people must select their own health insurance plan, which might explain the high percentage of switchers among the people between 18 and 40 years within the general population.

### Further research

Our results provide insight into switching rates for different groups ten years after the introduction of managed competition in the Netherlands. However, in the design of the current study, underlying mechanisms were not included. Therefore, future studies should ideally include attributes that might explain these differences, such as search behaviour, perceived barriers and possible switching costs. To explain the possible benefits of staying at the current insurer, satisfaction and loyalty are recommended for inclusion in further research to facilitate a better understanding of the differences in the switching rates. As earlier research indicated, group contracts, and having a supplementary insurance, play an important role in the consideration to switch health insurer as well [44, 45]. It is advised to assess whether consumers apply changes in supplementary insurance or group contracts without switching from insurer to insurer. Second, our study examined only the actual switching rates. High rates of intentional switchers can also send a clear signal to health insurers as the actual switching rates themselves [18]. Therefore, examining the people who intended to switch but refrained in the end, might provide an explanation for the low percentage of switchers among specific groups in future research. Third, to conclude, it can be recommended to examine switching behavior in a longitudinal setting, as this makes a contribution to the current body of literature by providing insight into the influence of life events, for instance being diagnosed with a chronic condition, on health insurance decision making.

### Implications for policy

Yet, it is unclear if the switching rates found in our study are sufficient to create enough competition between health insurers [17, 18]. It is important to mention that low switching rates on the health insurance market are not necessarily related to problems with the accessibility of care or a lack of consumer choice [46]. After all, a low switching rate may also be the result of a perfectly functioning health insurance market or a high level of satisfaction among the insured, which decreases their propensity to switch [46]. Also, it can be argued that high switching rates are not a favourable alternative, since this might increase the expenditures of health insurers. Based on the current research, we could not conclude if the safeguards mentioned before worked as intended to guarantee equal accessibility.

However, a switching percentage of 1% among the young chronically ill is remarkably low, and suggests that this specific group, or at least a part of it, might face barriers which may refuse them to switch. In addition, there might be the risk that a switching percentage of 1% is not sufficient to put pressure on the health insurers to meet the preferences of the young chronically ill. Additionally, it is likely that some of them would financially be better off by switching [47]. Although we did not examine how complicated decisions are for the insured, it can be reasoned that the health insurance system is complex [7, 47]. In 2016, the insured could choose from more than 61 policies for the basic insurance offered by 25 health insurers [48]. Comparing the different policies will be difficult when more policies are on the market for which not all providers are contracted. It is important to be sure, especially for the chronically ill, that their usual provider is contracted in the chosen insurance policy and to have insight into the selection criteria for contracting a health insurer. This demands effort, time, and the capacity to make the right decision.

The Netherlands’ Scientific Council for Government Policy (WRR) recently pointed out that taking action, for example, switching health insurer, not only depends upon people’s mental capacity, but also relies heavily on the ‘acting capacity’ [49]. Acting capacity refers to the ability to set a goal and take action [49]. This would indicate that the provision solely of clear information might thus not be sufficient to convince the insured to consider switching. Following the observation of the WRR, advice can be formulated in order to support switching health insurer in the system, in particular for the young chronically ill population. For instance, De Bekker et al. (2017) suggested that the insured should be encouraged to reflect on their health insurance every year, and should be stimulated to look for better options to make an informed choice [50]. Moreover, the government has been advised to provide more tailored information which would acknowledge the diversity between groups of people and the differences in the provision of services they require [51].

## Conclusions

Our study showed that the young chronically ill (18–39) switched the least of all groups. This observation suggests that this group might face difficulties or barriers which might prevent them from switching, or that they experience benefits from staying with their current insurer. Further research should therefore focus on unravelling the mechanisms which can explain the differences in switching rates between groups of insured. Government policy should focus on encouraging people to reflect on their current health plan every year. Providing tailored information about aspects other than price, such as the quality of care, should simplify the process of choosing a health insurer, especially for the young chronically ill.

## Supplementary information

**Additional file 1.** Original question about switching (translated from Dutch to English).

**Additional file 2.** Multivariate logistic regression with switching as the dependent variable for the general population.

## Data Availability

The dataset supporting the findings of this study is available on request from the authors and subject to approval by the program committees of both panels. The Dutch Health Care Consumer Panel (DHCCP) has a program committee, which supervises processing the data of the Dutch Health Care Consumer Panel and decides about the use of the data. This program committee consists of representatives of the Dutch Ministry of Health, Welfare and Sport, Health and Youth Care Inspectorate, Zorgverzekeraars Nederland (Association of Health Care Insurers in the Netherlands), the National Health Care Institute, the Federation of Patients and Consumer Organisations in the Netherlands, the Dutch Healthcare Authority and the Dutch Consumers Association. All research conducted within the Consumer Panel has to be approved by this program committee. The committee assesses whether a specific research fits within the aim of the Consumer Panel, that is strengthen the position of the health care user. Data are available upon request from Professor Judith D. de Jong, PhD (j.dejong@nivel.nl), project leader of the Dutch Health Care Consumer Panel. The program committee of the National Panel of people with a Chronic illness or Disability (NPCD) consists of representatives of the Dutch Ministry of Health, Welfare and Sport, Ministry of Social Affairs and Employment, Ieder (in) and The Netherlands institute for Social Research. Data are available upon request from Hennie Boeije, PhD (h.boeije@nivel.nl), project leader of the National Panel of people with a Chronic Illness.

## Abbrevations

OECD: Organisation for Economic Co-operation and Development
HIA: Health Insurance Act
DHCCP: Dutch Health Care Consumer Panel
NPCD: National Panel of the Chronically ill and Disabled
Nivel: The Netherlands Institute for Health Services Research
GP: General practitioner
