# Supplementary material for: Does the chronically ill population in the Netherlands switch their health insurer as often as the general population? Empirical evidence from a nationwide survey study
Source: BMC Health Serv Res. 2020 May 5;20:376. doi: 10.1186/s12913-020-05228-z (PMC7201544; doi:10.1186/s12913-020-05228-z)
Supplement: Supplementary file 1 — Additional file 1. Original question about switching (translated from Dutch to English). [file 12913_2020_5228_MOESM1_ESM.docx]

**Additional file 1**

**Original question about switching (translated from Dutch to English)**

**At the start of 2016, did you switch health insurer?**
❑ I did not switch, and not considered switching
❑ I did not switch, but considered switching
❑ I switched only for the basic insurance;
❑ I switched only for the additional insurance
❑ I switched for both the basic and additional insurance.
