# Supplementary material for: Does the chronically ill population in the Netherlands switch their health insurer as often as the general population? Empirical evidence from a nationwide survey study
Source: BMC Health Serv Res. 2020 May 5;20:376. doi: 10.1186/s12913-020-05228-z (PMC7201544; doi:10.1186/s12913-020-05228-z)
Supplement: Supplementary file 2 — Additional file 2. Multivariate logistic regression with switching as the dependent variable for the general population. [file 12913_2020_5228_MOESM2_ESM.docx]

**Additional file 2**

**Table S1: Multivariate logistic regression with switching as the dependent variable for the general population.**

|  |  | *.* | | |
| --- | --- | --- | --- | --- |
| **Switching (1=yes. 0=no)** |  | **Odds Ratio** | **P-value** | **Marginal Effect** |
| **Age** | 18-39 | Reference |  | 0.15 |
|  | 40-64 | 0.26 | 0.00 | 0.04 |
|  | 65 years and older | 0.24 | 0.00 | 0.04 |
| **Education** | Lower | Reference |  | 0.03 |
|  | Intermediate | 2.35 | 0.26 | 0.06 |
|  | Higher | 3.46 | 0.11 | 0.09 |
| **Perceived health condition** | Very good | Reference |  | 0.08 |
|  | Good | 0.64 | 0.21 | 0.06 |
|  | Bad | 0.81 | 0.71 | 0.07 |
| **Sex** | Male | Reference |  | 0.07 |
|  | Female | 0.92 | 0.81 | 0.07 |
| **Chronic illness** | No | Reference |  | 0.07 |
|  | Yes | 1.14 | 0.77 | 0.07 |
|  |  |  |  |  |
| **Constant** |  | 0.09 | 0.00 |  |
